# Integrated profiling identifies DXS253E as a potential prognostic marker in colorectal cancer

**DOI:** 10.1186/s12935-024-03403-4

**Published:** 2024-06-18

**Authors:** Pu Xing, Hao Hao, Jiangbo Chen, Xiaowen Qiao, Tongkun Song, Xinying Yang, Kai Weng, Yifan Hou, Jie Chen, Zaozao Wang, Jiabo Di, Beihai Jiang, Jiadi Xing, Xiangqian Su

**Affiliations:** 1 Key Laboratory of Carcinogenesis and Translational Research (Ministry of Education/Beijing), Department of Gastrointestinal Surgery IV, Peking University Cancer Hospital & Institute, No.52 Fucheng Road, Haidian District, Beijing, 100142 China; 2 Peking University Health Science Center, Beijing, 100191 China; 3 State Key Laboratory of Holistic Integrative Management of Gastrointestinal Cancers, Beijing Key Laboratory of Carcinogenesis and Translational Research, Department of Gastrointestinal Surgery IV, Peking University Cancer Hospital & Institute, No.52 Fucheng Road, Haidian District, Beijing, 100142 China

**Keywords:** Colorectal cancer, Bioinformatics, Prognosis, DXS253E, Glycolysis

## Abstract

**Background:**

Increasing evidence suggests that DXS253E is critical for cancer development and progression, but the function and potential mechanism of DXS253E in colorectal cancer (CRC) remain largely unknown. In this study, we evaluated the clinical significance and explored the underlying mechanism of DXS253E in CRC.

**Methods:**

DXS253E expression in cancer tissues was investigated using the Cancer Genome Atlas (TCGA) and Gene Expression Omnibus (GEO) databases. The Kaplan-Meier plot was used to assess the prognosis of DXS253E. The cBioPortal, MethSurv, and Tumor Immune Estimation Resource (TIMER) databases were employed to analyze the mutation profile, methylation, and immune infiltration associated with DXS253E. The biological functions of DXS253E in CRC cells were determined by CCK-8 assay, plate cloning assay, Transwell assay, flow cytometry, lactate assay, western blot, and qRT-PCR.

**Results:**

DXS253E was upregulated in CRC tissues and high DXS253E expression levels were correlated with poor survival in CRC patients. Our bioinformatics analyses showed that high DXS253E gene methylation levels were associated with the favorable prognosis of CRC patients. Furthermore, DXS253E levels were linked to the expression levels of several immunomodulatory genes and an abundance of immune cells. Mechanistically, the overexpression of DXS253E enhanced proliferation, migration, invasion, and the aerobic glycolysis of CRC cells through the AKT/mTOR pathway.

**Conclusions:**

We demonstrated that DXS253E functions as a potential role in CRC progression and may serve as an indicator of outcomes and a therapeutic target for regulating the AKT/mTOR pathway in CRC.

**Supplementary Information:**

The online version contains supplementary material available at 10.1186/s12935-024-03403-4.

## Introduction

Colorectal cancer (CRC) remains the major cause of tumor-related mortality in the world [1, 2]. Although significant progress in treatment has reduced recurrence and improved patient survival rates, the prognosis of CRC patients remains largely unknown. Therefore, it is necessary to gain a better understanding of oncogenic genes involved in CRC to develop therapeutic strategies for improving the long-term outcomes of CRC patients.

The TCGA and GEO databases are currently the most widely used public resource centers for oncology-related research [3–5]. These databases not only cover gene expression, methylation status, noncoding RNA, and other data, but, more importantly, contain clinical data and dynamically updated survival data. Bioinformatics analyses of TCGA and GEO data in cancer basic and clinical studies have facilitated the identification of numerous tumor markers, with some of these markers already being tested in clinical trials. Based on TCGA, Jin et al. identified a previously uncharacterized immunosuppressive tumor necrosis factor ligand molecule named CD70 that may serve as a possible chimeric antigen receptor target for immunotherapy in gliomas [6]. Additionally, another study listed in the TCGA’s database suggested that amplification of mouse double minute 4 (MDM4) is an important genetic change in the development of hepatocellular carcinoma (HCC), and a drug targeting this amplification has been undergoing clinical trials [7].

The solute carrier 10 (SLC10) family plays a crucial role in cell metabolism through the transportation of bile acids and many other substrates [8]. The SLC10 family consists of seven family members: SLC10A1 (NTCP), SLC10A2 (ASBT), SLC10A3 (DXS253E), SLC10A4 (P4), SLC10A5 (P5), SLC10A6 (SOAT), and SLC10A7 (P7). These molecules act as influx transporters of steroidal hormones and other specific agents [9]. NTCP, ASBT, and SOAT have been characterized as transport carriers, mainly used for the transport of bile acids and sulfated steroids [10, 11]. SLC10A4 is involved in exocytosis of neurotransmitters, vesicular storage, and mastocyte mediators; whereas SLC10A5 and SLC10A7 may participate in solute transport [12–14]. Due to crucial roles in molecular transport, alterations in the levels of SLC10 family members are associated with disturbances in metabolic and functional processes, which contribute to various diseases, including bile stasis, necrotizing enterocolitis, Barrett’s esophagus [15–17], skeletal dysplasia with multiple large joint dislocations, amelogenesis imperfecta, and short stature [18–20].

Moreover, previous studies have revealed the pivotal role of DXS253E in a series of cancers. Wang et al. found that DXS253E influences the chemotherapy resistance in polyploid cancer cells [21]. Recent studies have indicated that DXS253E is associated with the infiltration of immune cells in liver cancer [22]. Similarly, Wang et al. analyzed public databases and identified that DXS253E influences the immune microenvironment and is related to poor prognosis in patients with CRC [23]. However, the exact role of DXS253E in CRC remains to be further determined through the specific experiments.

In this study we evaluated the clinical significance and effects of DXS253E in CRC through TCGA and GEO, and with an experimental cohort. We found that high levels of DXS253E correlate with poor prognosis in CRC patients. Our bioinformatics analyses also show that the methylation of DXS253E is associated with the prognosis of CRC patients and that DXS253E levels correlate with immune cell infiltration. Moreover, we, for the first time, explored the function of DXS253E in vitro using cell culture experiments. We found that DXS253E enhances the malignant biological behavior and aerobic glycolysis through the AKT/mTOR pathway in CRC. Systematically investigating the role of DXS253E may facilitate the identification of novel therapeutic targets for CRC.

## Materials and methods

### DXS253E expression analysis

DXS253E mRNA expression levels were determined for 33 human cancers including CRC tissues based on TCGA and the GEO databases. The R programming language (version 3.6.3) was used to download, clean, and visualize the data. In detail, the ‘TCGAbiolinks’ (version 2.28.4) and ‘GEOquery’ (version 2.72.0) R packages were used to download RNA-sequencing and clinical data from TCGA and the GEO databases, respectively [24, 25]. Subsequently, all of the cancer RNA-sequencing data were merged into a single meta-cohort via ComBat algorithm from the ‘sva’ R package (version 3.48.0) to eliminate batch effects [26]. Following the combination of 33 datasets, we normalized the raw data using a log2(FPKM + 1) transformation. Probes were converted into corresponding gene symbols based on a GPL570 annotation file. Finally, DXS253E mRNA expression levels were filtered using the R package ‘tidyverse’ (version 2.0.0) and plotted using the ‘ggplot2’ R package (version 3.4.4) [27].

### Patients and tissue specimens

The Department of Gastrointestinal Surgery IV, Peking University Cancer Hospital & Institute, provided eight matched sets of CRC and normal tissues from patients who had surgical resection between September 2009 and October 2011. Table S1 contains detailed clinical information. Written informed consent was taken from each patient before sample collection. The Research Ethics Committee of Peking University Cancer Hospital & Institute approved and supervised this study (2021KT134).

### Immunohistochemistry

Samples of CRC patients and nearby healthy tissues were preserved in 4% paraformaldehyde (PFA), embedded in paraffin, cut into 4 μm slices, and then heated at 65 °C for two hours. After heat-induced epitope retrieval, the slides were incubated with DXS253E antibody (1:100, Cat #19,909-1-AP, Proteintech) overnight at 4 °C, and subsequently exposed to an anti-rabbit antibody for 40 min and stained with fresh 3,3′-Diaminobenzidine substrate within a controlled reaction time of 1–2 min. Next, sections were counterstained with hematoxylin, rinsed to blue, dehydrated, and sealed.

### Survival analysis

The association between DXS253E expression and the prognosis of CRC patients was evaluated by Kaplan-Meier analysis with the log-rank test. The results were visualized using the R survminer package. The effect of clinical variables on patient outcome was assessed using univariate and multivariate Cox regression analyses. Tumor stage correlation was analyzed using the R ggplot2 and stats packages.

### DNA methylation analysis

The status of DXS253E methylation in CRC was investigated using the MethSurv web-based tool (https://biit.cs.ut.ee/methsurv/). Moreover, the prognostic value of DXS253E methylation levels in CRC patients was evaluated using MethSurv and its built-in database.

### Differentially expressed gene analysis

The Differentially expressed gene analysis (DEGs) between DXS253E high- and DXS253E low-expression groups were identified using the R Bioconductor package DESeq2 (https://www.bioconductor.org/). Then, the DEGs were drawn as volcano maps using the R ggplot2 package.

### Gene function enrichment analysis

The R Bioconductor clusterProfiler package was used to evaluate our Gene Ontology (GO), Kyoto Encyclopedia of Genes and Genomes (KEGG) analyses and GSEA with the identified DEGs. An adjusted *P* value < 0.05 was regarded as statistically significant.

### Immune correlation analysis

The ssGSEA method provided by the GSVA package was used to determine the infiltration degree of immune cells with notable DXS253E expression levels [28]. Given that the expression matrix is in the format of FPKM (Fragments Per Kilobase of transcript per Million mapped reads), the kcdf parameter was set to “Gaussian” [28]. We downloaded markers for 24 types of immune cells following the Bindea et al. protocol [29]. Subsequently, a Pearson’s correlation analysis was conducted to investigate the relationship between DXS253E expression levels and the infiltration of immune cells based on a previous study [30]. An absolute correlation coefficient value greater than 0.3, accompanied by a *P* value less than 0.05, was considered significant for these correlations [31]. Additionally, the association between DXS253E and immune-related genes was examined using the Sangerbox toolkit (http://www.sangerbox.com/) [32].

### Cell lines and cell culture

All human CRC cell lines (LoVo, HCT116, RKO, SW480, and SW620) and a healthy human intestinal epithelial cell line (NCM460) were obtained from American Type Culture Collection. Cells were cultured in Dulbecco’s modified Eagle’s medium (DMEM) high glucose medium (HyClone, Logan, UT, USA), which was supplemented with 10% fetal bovine serum and 1% penicillin/streptomycin at 37 °C with 5% CO_2_.

### Western blot analysis

Primary antibodies were diluted to appropriate concentrations: DXS253E (1:1,000, Cat #19,909-1-AP, Proteintech), p-mTOR (1:,1000, Cat #2971, Cell Signaling Technology), mTOR (1:2,000, Cat #2983, Cell Signaling Technology), p-AKT (1:1,000; Cat #9271, Cell Signaling Technology), AKT (1:2,000; Cat #9272, Cell Signaling Technology), PKM2 (1:1,000, Cat #4053, Cell Signaling Technology), HK2 (1:2,000, Cat #2867, Cell Signaling Technology), GLUT1 (1:2,000, Cat #21,829-1-AP, Proteintech), and LDHA (1:1,000, Cat #2012, Cell Signaling Technology). β-actin (1:5,000; Cat #A1978, Sigma-Aldrich) was used as a control. Protein band densities were quantified using ImageJ software.

### Quantitative real-time PCR

Total RNA from CRC tissues and cells was isolated using Trizol (Invitrogen, Waltham, MA, USA) and then reversely transcribed into cDNA with a reverse transcription kit (Promega, Madison, WI, USA). Thereafter, qRT-PCR was carried out using a SYBR-Green Master kit (Cat# 147,100, TOYOBO, Japan). The primers used for amplification are presented in Table S2.

### CCK-8 and colony formation assay

CRC cell proliferation was assessed using CCK-8 assays (Dojindo, Japan). The cells were inoculated into 96-well plates at a density of 5,000 cells per well with complete medium. After being cultured for the indicated time, 10 µL of CCK-8 solution was added to each well and incubated for an additional 2 h at 37 °C. Spectrometric absorbance values were then measured at 450 nm. To assess colony formation ability, the cells were plated at a density of 500 cells per well and incubated in 6-well plates for 12 days. Colonies were stabilized with 4% PFA, and stained with 0.1% crystal violet.

### Transwell assays

Cell migration and invasion assays were performed using 24-well Corning® Costar® Transwell chambers (NY, USA). The cells were resuspended in serum-free medium and inoculated into the Transwell chambers with or without Corning® Matrigel and then we conducted migration or invasion assays, respectively. Complete medium was added to the lower chambers. After incubation for 24 h, migrated cells were treated with 4% PFA and stained with 0.1% crystal violet. Images were taken under a microscope and the number of migrating cells was counted.

### Lactate production and detection of reactive oxygen species (ROS)

Cells were transfected with plasmids containing DXS253E and cultured for 48 h in complete medium. The culture medium was harvested to determine lactate production with a lactic acid assay kit (Nanjing Jiancheng, China). Cells were incubated with 2 µM diacetyldichlorofluorescein (Solarbio, China) at 37 °C for 30 min. After incubation, cells were washed with 1 ml of phosphate buffered saline three times. Fluorescence intensity of the cells was then recorded using a flow cytometer.

### Statistical analysis

All statistical analyses were conducted using R (version 3.6.3). DXS253E mRNA expression levels across various cancers were calculated using a Wilcoxon rank-sum test based on TCGA and GEO databases. Student’s t tests were conducted to determine mRNA expression differences between the CRC and normal samples in our cohort. The correlation between clinicopathologic features and the level of DXS253E was analyzed with a Wilcoxon rank-sum test and logistic regression. For in vitro experiments, comparisons between groups were detected using Student’s t tests.

## Results

### DXS253E is highly expressed in a series of cancers including CRC

**Fig. 1:**
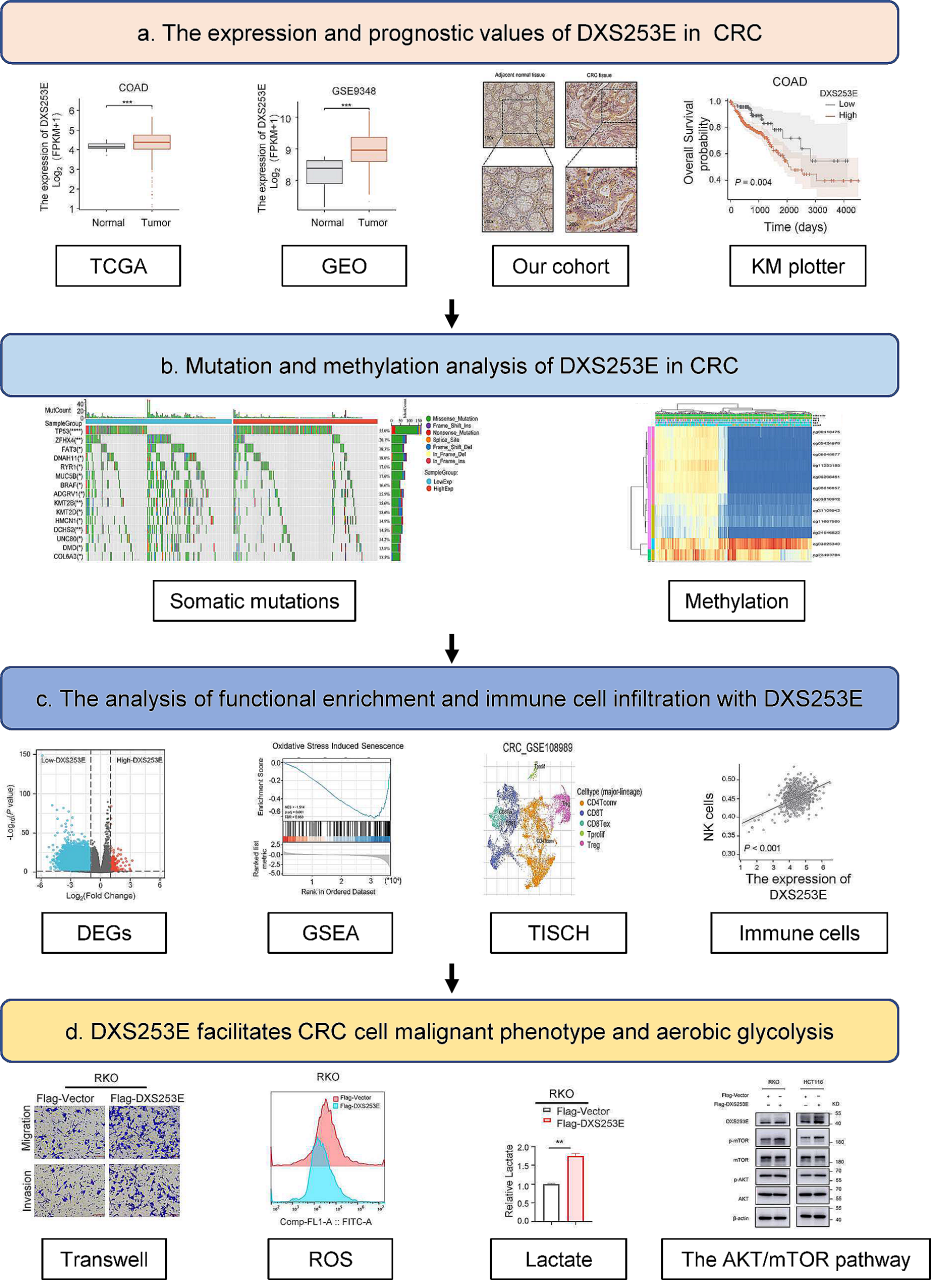
The analysis flowchart of this study

**Fig. 2:**
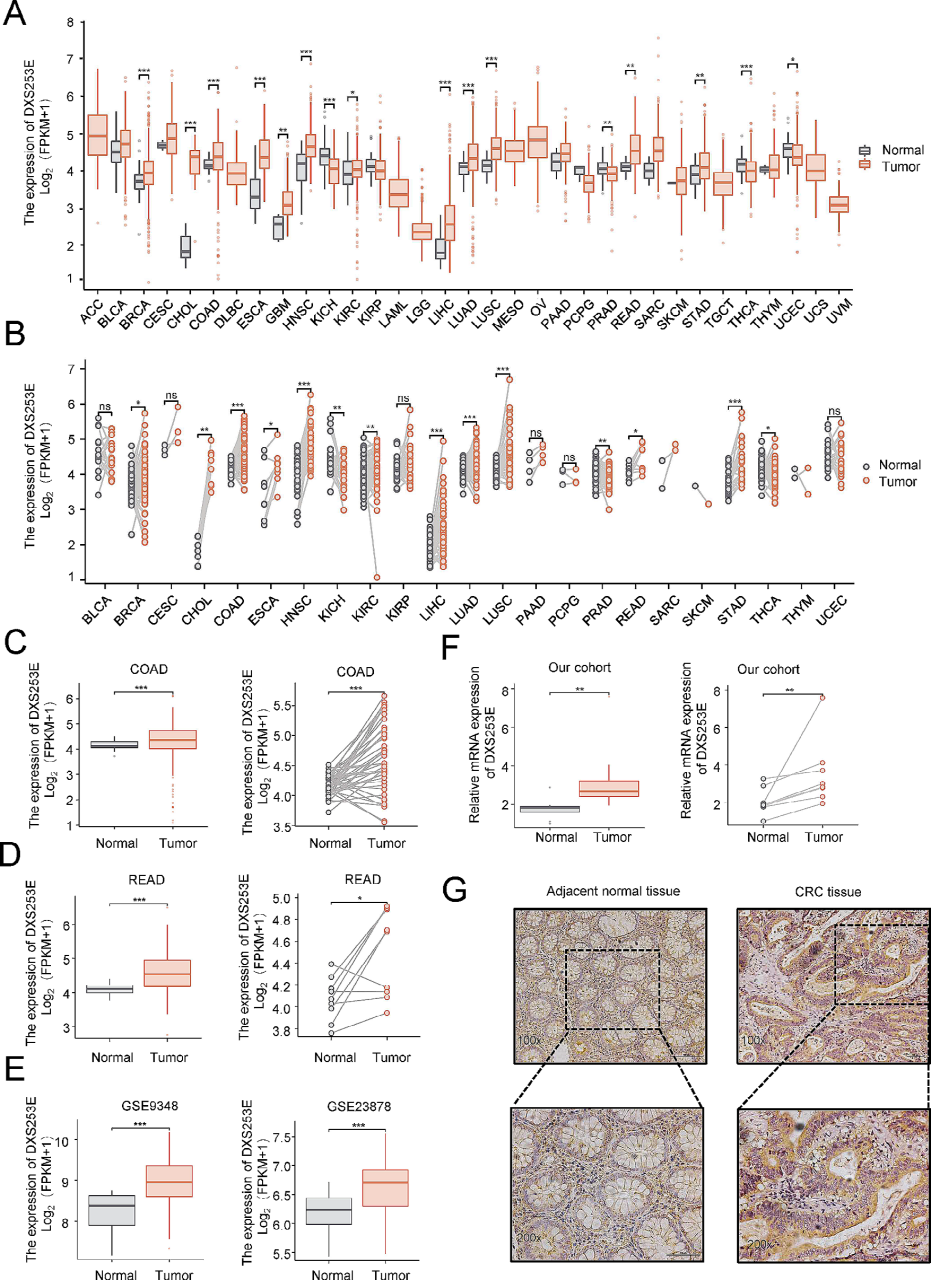
DXS253E expression is increased in different types of tumors including colorectal cancer (CRC). **A** Pan-cancer analysis of DXS253E expression levels in human tumors according to the Cancer Genome Atlas (TCGA) dataset. **B** Expression of DXS253E in cancer and para-cancer paired tissues based on TCGA dataset. **C, D** Comparison of the expression levels of DXS253E in colon adenocarcinoma (COAD) (C) or rectum adenocarcinoma (READ) (D) and normal tissues from TCGA’s database. **E** Gene Expression Omnibus database (GEO) analysis of DXS253E expression in CRC tissues. **F** qRT-PCR assay of DXS253E mRNA expression levels in eight pairs of CRC and adjacent tissues from our experimental cohort. **G** Representative images of DXS253E expression in CRC tissues and matched normal tissues. Original magnifications 100× and 200× (inset panels). *P < 0.05, **P < 0.01, ***P < 0.001, ns, no significance

A flowchart containing the detailed procedures of this study is presented in Fig. 1. First, our pan-cancer analyses based on TCGA’s database suggest that high DXS253E expression is involved in a series of cancers, such as breast invasive carcinoma (BRCA) and cholangiocarcinoma (CHOL) (Fig. 2A, B). The expression level of DXS253E is notably higher in colon adenocarcinoma (COAD) and rectum adenocarcinoma (READ) samples than in normal tissues (Fig. 2C, D). Moreover, we identified high DXS253E expression in CRC tissues from the GEO datasets GSE9348 and GSE23878 compared with corresponding controls (Fig. 2E). To verify our database results, we determined DXS253E expression in CRC and adjacent normal tissues using quantitative real-time PCR (qRT-PCR) and immunohistochemistry from our CRC cohort. Our results show a markedly increased expression of DXS253E in CRC tissues compared with adjacent normal tissues (Fig. 2F, G).

### High DXS253E expression indicates worse prognosis for CRC patients

**Fig. 3:**
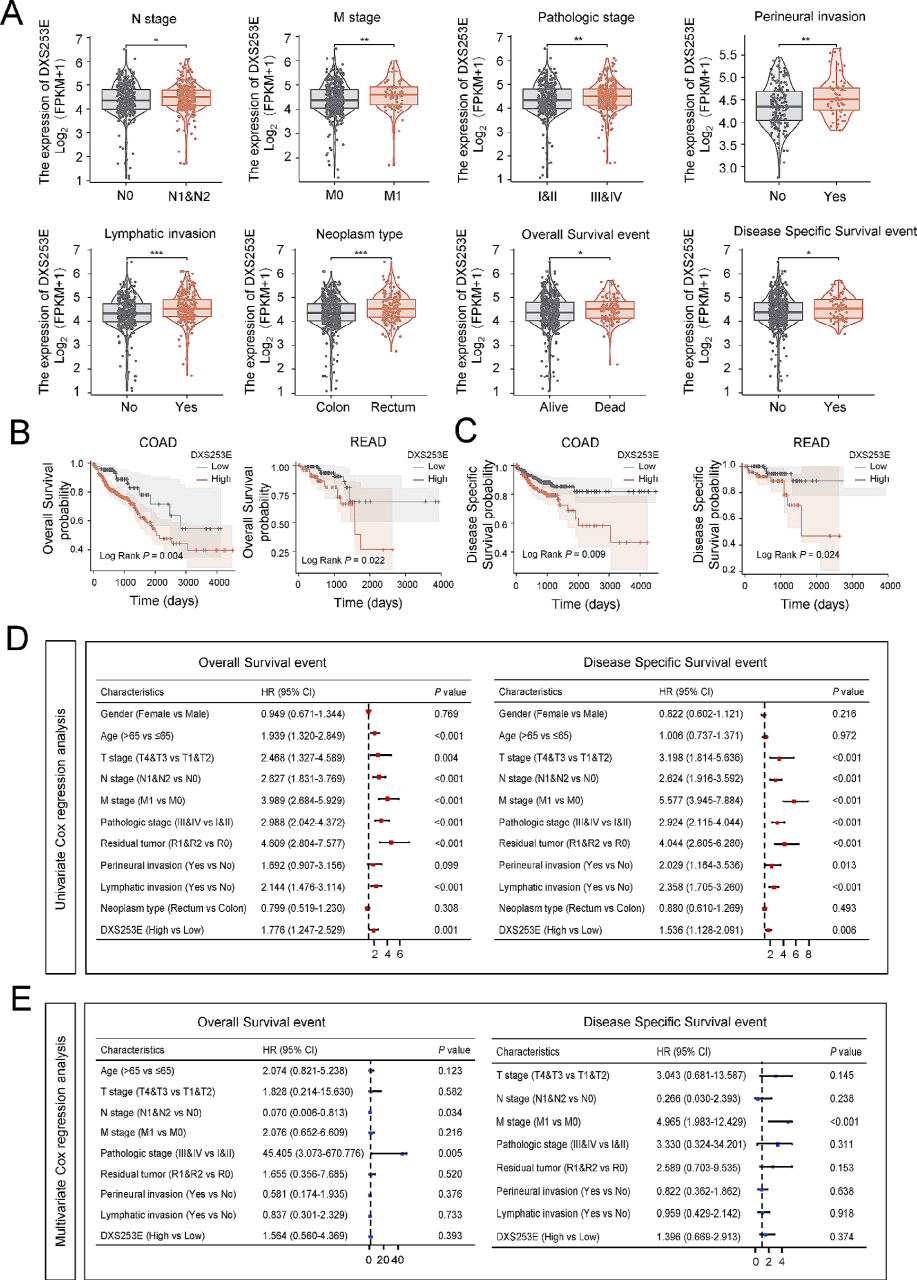
High DXS253E expression indicates aggressive clinical features and poor prognosis for CRC patients. **A** Association between DXS253E expression and clinical characteristics. **B, C** Effects of DXS253E level on overall survival (OS) and disease-specific survival (DSS) in COAD and READ. **D** Forest plot of univariate Cox regression analysis of DXS253E mRNA expression with OS and DSS in CRC with different clinicopathological features. **E** Forest plot of multivariate Cox regression analysis of DXS253E mRNA expression with OS and DSS in CRC with different clinicopathological features. *P < 0.05, **P < 0.01, ***P < 0.001

We evaluated the mRNA levels of DXS253E in different clinical categories to determine the correlation between DXS253E expression and clinicopathological features in CRC patients within TCGA’s database. Our findings indicated that the expression of DXS253E is significantly associated with N stage (*P* = 0.003), M stage (*P* = 0.002), pathologic stage (*P* = 0.001), perineural invasion (*P* = 0.039), lymphatic invasion (*P* < 0.001), neoplasm type (*P* = 0.038), overall survival (OS) (*P* = 0.010), and disease-specific survival (DSS) events (*P* = 0.040) in CRC patients (Table S3, Fig. 3A). However, no significant correlations were observed between DXS253E and other clinicopathological variables, such as T stage, gender, age, and race (Table S3). Collectively, these data suggest that high DXS253E expression is related to more lymph node metastases, more perineural invasion, higher pathologic, N, and M stage occurrences, and poor prognosis in CRC patients.

Kaplan-Meier plots were used to analyze the relationship between DXS253E expression and prognosis of patients with CRC in the TCGA’s database. We found that higher DXS253E expression has a significant association with poor OS in COAD (*P* = 0.004) and READ (*P* = 0.022) (Fig. 3B). Similarly, DSS analysis data showed that higher DXS253E expression also correlated with poor prognosis in COAD (*P* = 0.009) and READ (*P* = 0.024) (Fig. 3C).

We also performed univariate Cox analysis in TCGA’s database to further investigate the influence of DXS253E expression on CRC prognosis, and we identified that DXS253E level was a significant predictor for OS (hazard ratio [HR]: 1.776; 95% confidence interval [CI]: 1.247–2.529; *P* = 0.001) and DSS (HR: 1.536; 95% CI: 1.128–2.091; *P* = 0.006) (Fig. 3D; Tables 1 and 2). Additionally, age (*P* < 0.001), T stage (*P* = 0.004), N stage (*P* < 0.001), M stage (*P* < 0.001), pathologic stage (*P* < 0.001), residual tumor (*P* < 0.001), and lymphatic invasion (*P* < 0.001) were all significant variables in the univariate analysis of OS. Furthermore, T stage (*P* < 0.001), N stage (*P* < 0.001), M stage (*P* < 0.001), pathologic stage (*P* < 0.001), residual tumor (*P* < 0.001), perineural invasion (*P* = 0.013), and lymphatic invasion (*P* < 0.001) were also found to be significant clinical features in the univariate analysis of DSS.

**Table 1:** Univariable and multivariable analysis for OS in CRC patients with TCGA cohort

Variables	Univariate analysis			Multivariate analysis	
	HR (95% CI)	*P* value		HR (95% CI)	*P* value
Gender (Female vs. Male)	0.949 (0.671–1.344)	0.769			
Age (> 65 vs. ≤ 65)	1.939 (1.320–2.849)	**< 0.001**		2.074 (0.821–5.238)	0.123
T stage (T4&T3 vs. T1&T2)	2.468 (1.327–4.589)	**0.004**		1.828 (0.214–15.630)	0.582
N stage (N1&N2 vs. N0)	2.627 (1.831–3.769)	**< 0.001**		0.070 (0.006–0.813)	**0.034**
M stage (M1 vs. M0)	3.989 (2.684–5.929)	**< 0.001**		2.076 (0.652–6.609)	0.216
Pathologic stage (III&IV vs. I&II)	2.988 (2.042–4.372)	**< 0.001**		45.405 (3.073-670.776)	**0.005**
Residual tumor (R1&R2 vs. R0)	4.609 (2.804–7.577)	**< 0.001**		1.655 (0.356–7.685)	0.520
Perineural invasion (Yes vs. No)	1.692 (0.907–3.156)	0.099		0.581 (0.174–1.935)	0.376
Lymphatic invasion (Yes vs. No)	2.144 (1.476–3.114)	**< 0.001**		0.837 (0.301–2.329)	0.733
Neoplasm type (Rectum vs. Colon)	0.799 (0.519–1.230)	0.308			
DXS253E (High vs. Low)	1.776 (1.247–2.529)	**0.001**		1.564 (0.560–4.369)	0.393

HR: hazard ratio; CI: confidence interval. *P*-values in bold were statistically significant

**Table 2:** Univariable and multivariable analysis for DSS in CRC patients with TCGA cohort

Variables	Univariate analysis			Multivariate analysis	
	HR (95% CI)	*P* value		HR (95% CI)	*P* value
Gender (Female vs. Male)	0.822 (0.602–1.121)	0.216			
Age (> 65 vs. ≤ 65)	1.006 (0.737–1.371)	0.972			
T stage (T4&T3 vs. T1&T2)	3.198 (1.814–5.636)	**< 0.001**		3.043 (0.681–13.587)	0.145
N stage (N1&N2 vs. N0)	2.624 (1.916–3.592)	**< 0.001**		0.266 (0.030–2.393)	0.238
M stage (M1 vs. M0)	5.577 (3.945–7.884)	**< 0.001**		4.965 (1.983–12.429)	**< 0.001**
Pathologic stage (III&IV vs. I&II)	2.924 (2.115–4.044)	**< 0.001**		3.330 (0.324–34.201)	0.311
Residual tumor (R1&R2 vs. R0)	4.044 (2.605–6.280)	**< 0.001**		2.589 (0.703–9.535)	0.153
Perineural invasion (Yes vs. No)	2.029 (1.164–3.536)	**0.013**		0.822 (0.362–1.862)	0.638
Lymphatic invasion (Yes vs. No)	2.358 (1.705–3.260)	**< 0.001**		0.959 (0.429–2.142)	0.918
Neoplasm type (Rectum vs. Colon)	0.880 (0.610–1.269)	0.493			
DXS253E (High vs. Low)	1.536 (1.128–2.091)	**0.006**		1.396 (0.669–2.913)	0.374

HR: hazard ratio; CI: confidence interval. *P*-values in bold were statistically significant

Finally, we conducted a multivariate Cox regression analysis using the significant factors identified in our univariate analysis (Fig. 3E). Our results demonstrate that advanced pathologic stage (*P* = 0.005) and advanced M stage (*P* < 0.001) are independent prognostic variables of poor prognosis for OS and DSS, respectively.

### Genetic and epigenetic alterations of the DXS253E gene in CRC

**Fig. 4:**
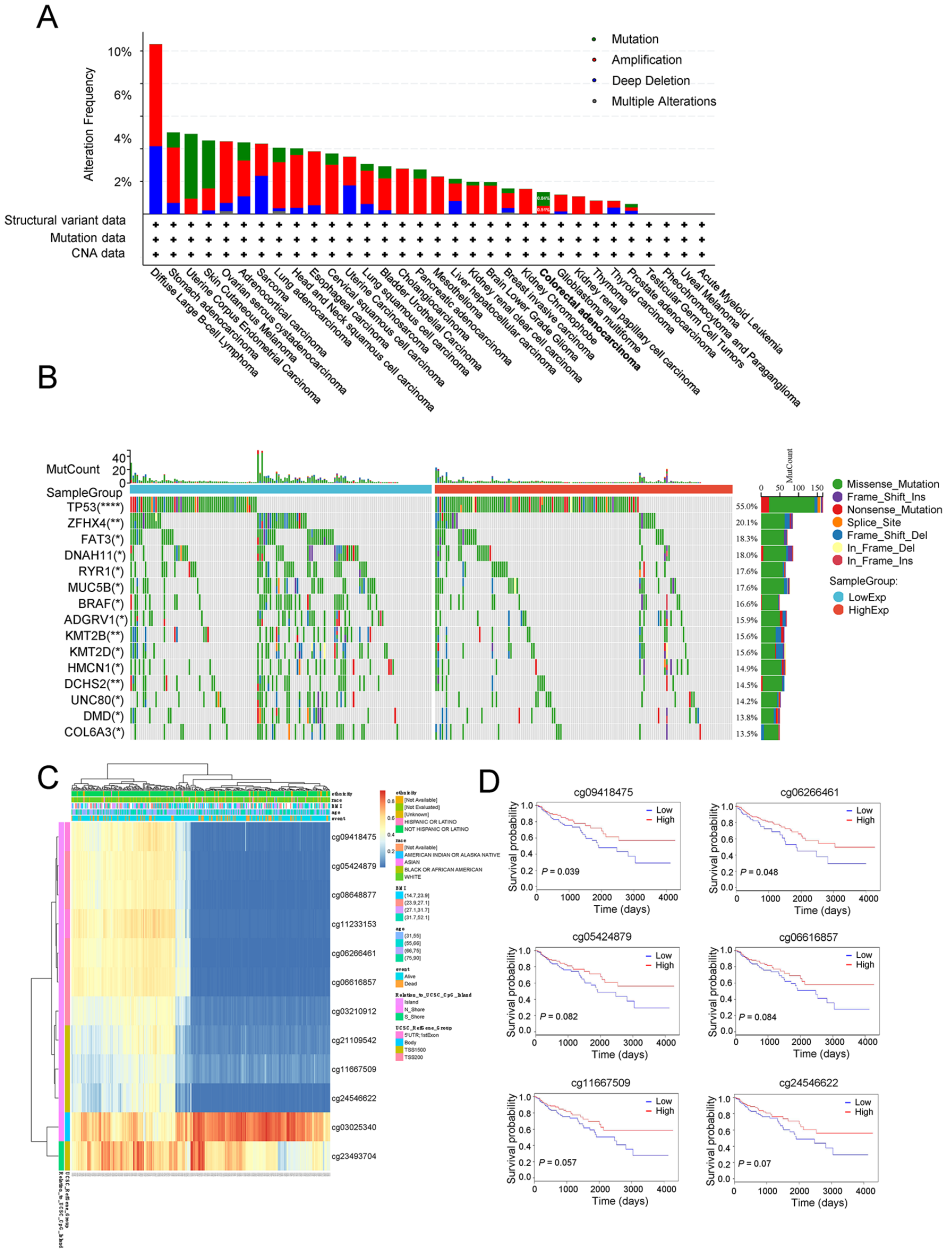
Mutation and methylation analysis of DXS253E in CRC. **A** Alteration frequency of the DXS253E gene across various cancers analyzed using the cBioPortal web resource. **B** Differential somatic mutations identified in CRC between low and high DXS253E expression groups. **C** Correlation between DXS253E mRNA expression level and methylation level. **D** Kaplan-Meier survival curves showing six methylation sites in the DXS253E gene

To explore mutations in the DXS253E gene in a series of cancers, we analyzed the mutation status of SCL10A3 using the Web cBioPortal platform (https://www.cbioportal.org/) with TCGA’s pan-cancer database. The highest alteration frequency of the DXS253E gene (10.42%) was identified in diffuse large B cell lymphoma patients with amplification and deep deletion as the primary alterations (Fig. 4A). Our analysis results showed a DXS253E gene mutation frequency of 0.84%, and an amplification frequency of 0.51% for DXS253E gene in CRC (Fig. 4A).

Additionally, we performed exon missense mutation analysis in high and low DXS253E expression groups using CRC TCGA data. The top 15 notably different somatic mutations occurred in the following proteins: TP53, ZFHX4, FAT3, DNAH11, RYR1, MUC5B, BRAF, ADGRV1, KMT2B, KMT2D, HMCN1, DCHS2, UNC80, DMD, and COL6A3 (Fig. 4B). Our results uncovered a remarkably high mutation frequency for the TP53 gene in the DXS253E high-expression group. These mutated genes may prove valuable for determining tumor progression and therapeutic response in CRC patients.

DNA methylation was investigated to determine epigenetic effects, according to TCGA’s database. DNA methylation levels of the gene encoding DXS253E and the prognostic value of the CpG islands in this gene were determined using the web’s MetSurv tool. Twelve methylated CpG islands were identified as being correlated with levels of DXS253E gene methylation, in particular cg09418475 and cg05424879 (Fig. 4C). Furthermore, Kaplan-Meier survival analysis suggested that two CpG sites (cg09418475, *P* = 0.039 and cg06266461, *P* = 0.048) exhibited high methylation levels that were significantly correlated with favorable OS of CRC. We observed that following four other DXS253E CpG sites were borderline significantly related to the OS of CRC patients: cg05424879 (*P* = 0.082), cg06616857 (*P* = 0.084), cg11667509 (*P* = 0.057), and cg24546622 (*P* = 0.07) (Fig. 4D).

### Identification and functional enrichment analysis of DEGs related to DXS253E

**Fig. 5:**
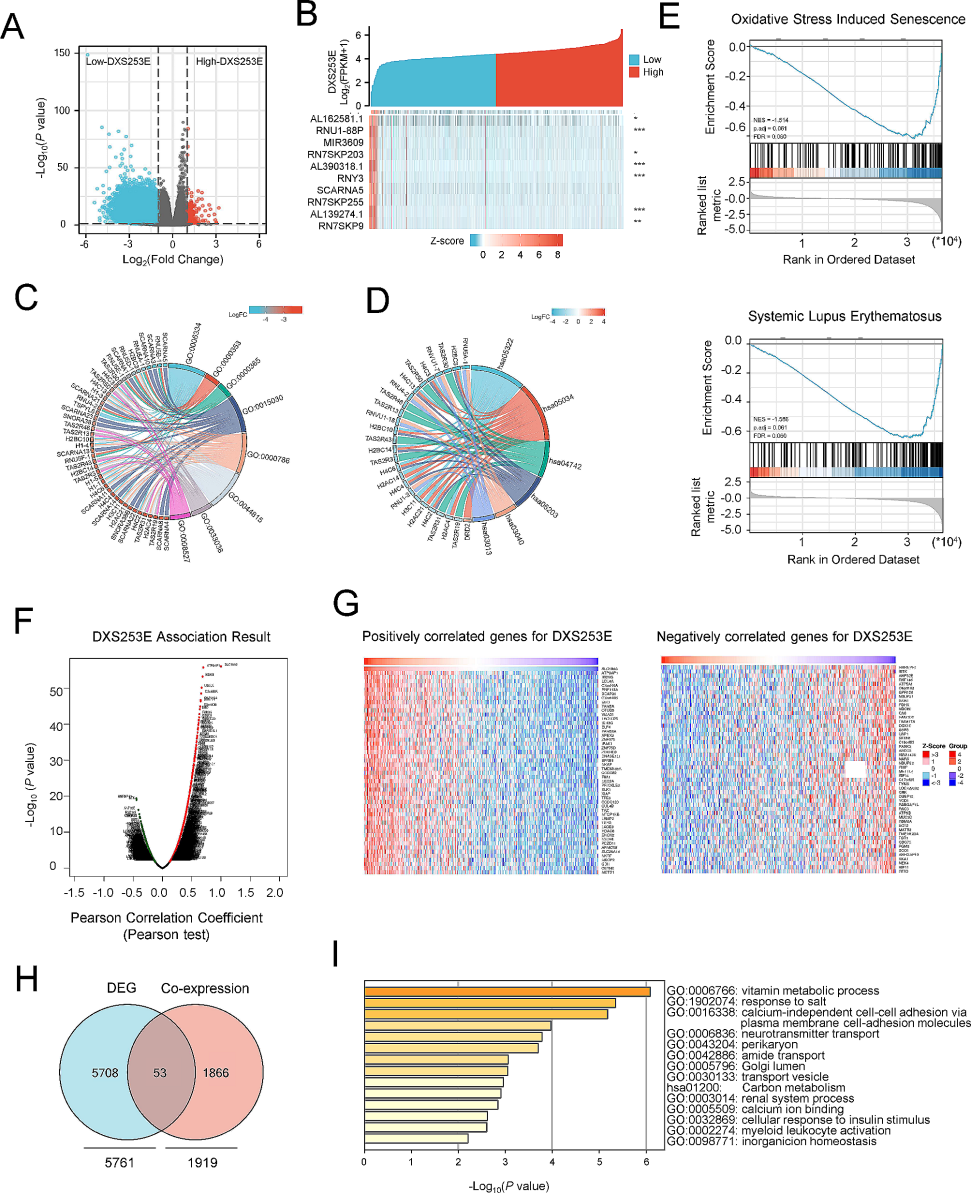
Functional enrichment analysis of differentially expressed genes (DEGs) according to DXS253E expression level in CRC. **A** Volcano plot for DEGs between low DXS253E and high DXS253E expression groups. **B** Heatmap showing the top 10 DEGs between low and high DXS253E-expression groups. **C** GO enrichment analysis of DXS253E-associated DEGs. **D** KEGG enrichment analysis of DXS253E-associated DEGs. **E** GSEA of relevant signaling pathways in CRC tissues based on DXS253E-related DEGs. **F** Volcano plot of co-expressed genes correlated with DXS253E expression using the LinkedOmics web resource. **G** Heatmaps of the top 50 genes that are positively or negatively associated with DXS253E. **H** Venn diagram of the number of intersections between DXS253E DEGs and co‐expressed genes in CRC. **I** Enrichment analysis of overlapping genes analyzed by the Metascape web resource

We compared DEGs between low-expression and high-expression groups of DXS253E in CRC based on TCGA’s database, to further investigate DXS253E mechanisms in CRC. A total of 5,761 representative DEGs (*P* < 0.05, |log2 FC |≥ 1) were identified in the DXS253E-high group, with 215 upregulated and 5,546 downregulated genes (Fig. 5A, Table S4). The top 10 DEGs were AL162581.1, RNU1-88P, MIR3609, RN7SKP203, AL390318.1, RNY3, SCARNA5, RN7SKP255, AL139274.1, and RN7SKP9 (Fig. 5B).

Gene ontology (GO) enrichment analysis revealed that the DEGs we identified were enriched with several GO terms. These included nucleosome assembly; mRNA trans splicing, via spliceosome; formation of quadruple SL/U4/U5/U6 snRNP; Cajal body; nucleosome; DNA packaging complex; bitter taste receptor activity; and taste receptor activity (Fig. 5C, Table S5). Additionally, analyses at KEGG pathway database suggested that these DEGs are significantly enriched in systemic lupus erythematosus, alcoholism, taste transduction, viral carcinogenesis, spliceosome, and RNA transport pathways (Fig. 5D, Table S6). Subsequently, we conducted GSEA analyses using GSEA software. The results indicated that DXS253E predominantly and positively regulated the processes of oxidative stress-induced senescence and systemic lupus erythematosus (Fig. 5E, Table S7).

We next used the LinkedOmics web portal (https://www.linkedomics.org/) to identify genes correlated with DXS253E in CRC. Pearson correlation analysis revealed that a total of 1,919 co-expressed genes were remarkably associated with DXS253E in CRC (FDR < 0.05, *P* < 0.05, and |cor.| ≥ 0.2) (Fig. 5F, Table S8). The top 50 positively (*r* > 0) and negatively (*r* < 0) correlated genes that we found were included in the heat maps of this cohort (Fig. 5G).

Next, the 5,761 DXS253E-associated DEGs from TCGA’s database that we identified and the 1,919 significantly co-expressed genes found with LinkedOmics were selected to determine how many occurred in both dataset results. Among them, 53 genes overlapped and these were selected for further functional analyses (Fig. 5H, Table S9). We then performed a combined GO/KEGG analysis through the Metascape tool (https://metascape.org/) to explore biological functions of the 53 overlapping genes. This enrichment analysis suggested that the following three functions were most significantly enriched in the 53 genes: vitamin metabolic processes, response to salt, and calcium-independent cell-cell adhesion via plasma membrane cell-adhesion molecules (Fig. 5I, Table S10).

### DXS253E expression is associated with immune-related genes and immune cell infiltration

**Fig. 6:**
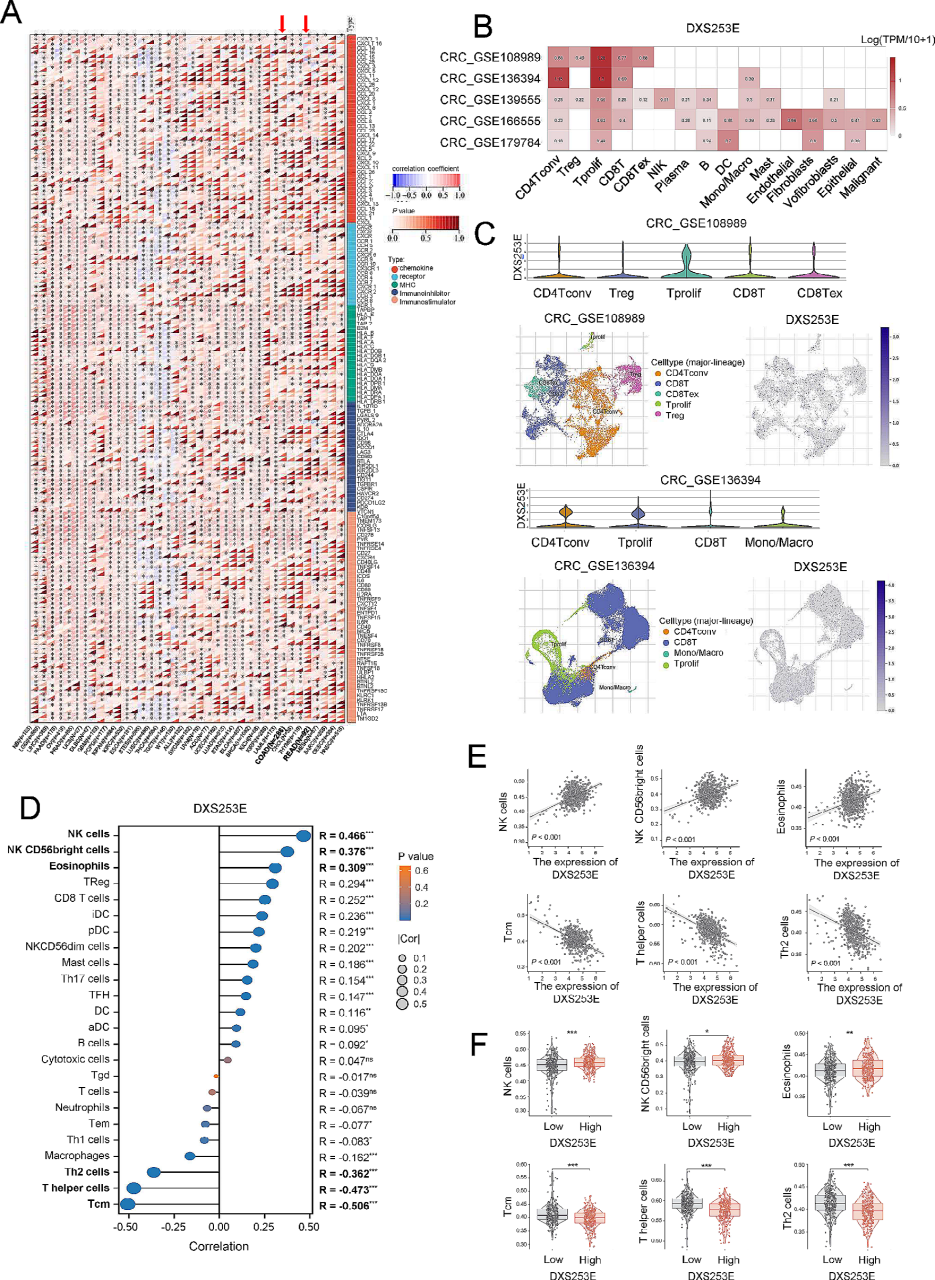
DXS253E expression is associated with multiple immune-related genes, and immune cell infiltration occurs in CRC. **A** Correlation of DXS253E with immunomodulatory genes in pan-cancer. **B** Heatmap of DXS253E expression with various tumor microenvironment cells in five independent datasets from the Tumor Immune Single-cell Hub (TISCH) database. **C** DXS253E expression in immune cells according to the Gene Expression Omnibus (GEO) GSE108989 and GSE136394 datasets. **D** Correlation between DXS253E and immune cell infiltration. **E, F** Relationship of DXS253E expression with the infiltration level of NK, NK CD56bright, eosinophil, Tcm, T helper, and Th2 cells using scatter plots (E) and box plots (F). *P < 0.05, **P < 0.01, ***P < 0.001

Given the known essential influence of the tumor microenvironment (TME) on patient prognosis and treatment decisions [33, 34], we investigated the association between DXS253E and the TME. First, in pan-cancer dataset, we determined the correlation of DXS253E with immunomodulatory genes which encoded chemokines, receptors, MHC, immuno-inhibitors, and immuno-stimulators. Our results showed that DXS253E was significantly correlated with various immune-associated genes in multiple cancers, including COAD and READ (Fig. 6A).

Immune cells play an important role in orchestrating the TME. Increasing evidence shows that single-cell RNA sequencing (scRNA-seq) is very useful for analyzing the TME and immune cell infiltration [35, 36]. In this study, we found five independent CRC scRNA-seq entries in the Tumor Immune Single-cell Hub database (http://tisch.comp-genomics.org/home/) at the time of our research (CRC_GSE108989, CRC_GSE136394, CRC_GSE139555, CRC_GSE166555, and CRC_GSE179784). We then explored DXS253E expression levels at the single-cell level in various cells of the TME. Our findings indicated that DXS253E was highly expressed in immune and endothelial cells (Fig. 6B). Specifically, in the CRC_GSE108989, higher levels of DXS253E expression were found in Tprolif cells. In the CRC_GSE136394 dataset, DXS253E was mainly distributed in conventional CD4Tconv and Tprolif cells (Fig. 6C).

Tumors can modulate the immune cells infiltration and immune response through specific molecules in the TME [37]. Therefore, we aimed to investigate whether the DXS253E level in tumors could impact the recruitment of immune cells. Our analysis using TCGA datasets demonstrated that DXS253E was significantly linked to the infiltration of multiple immune cells, including NK, NK CD56bright, eosinophil, Tcm (a subgroup of memory CD8^+^T cells), T helper, and Th2 cells (a subgroup of CD4^+^T cells) (all *P* < 0.05, |R| ≥ 0.3, Fig. 6D) cells. Our scatter plot illustrates that the expression of DXS253E was positively connected with NK, NK CD56bright, and eosinophil cells (*P* < 0.001), but was negatively connected with Tcm, T helper, and Th2 cells (*P* < 0.001) (Fig. 6E). Similarly, NK, NK CD56bright, eosinophil, Tcm, T helper, and Th2 cells showed differential infiltration when samples were subdivided according to DXS253E expression levels in CRC (Fig. 6F). Thus, DXS253E expression levels are significantly associated with immune-related genes and immune cell infiltration in CRC.

### DXS253E overexpression promotes malignant biological behavior of and aerobic glycolysis in CRC cells

**Fig. 7:**
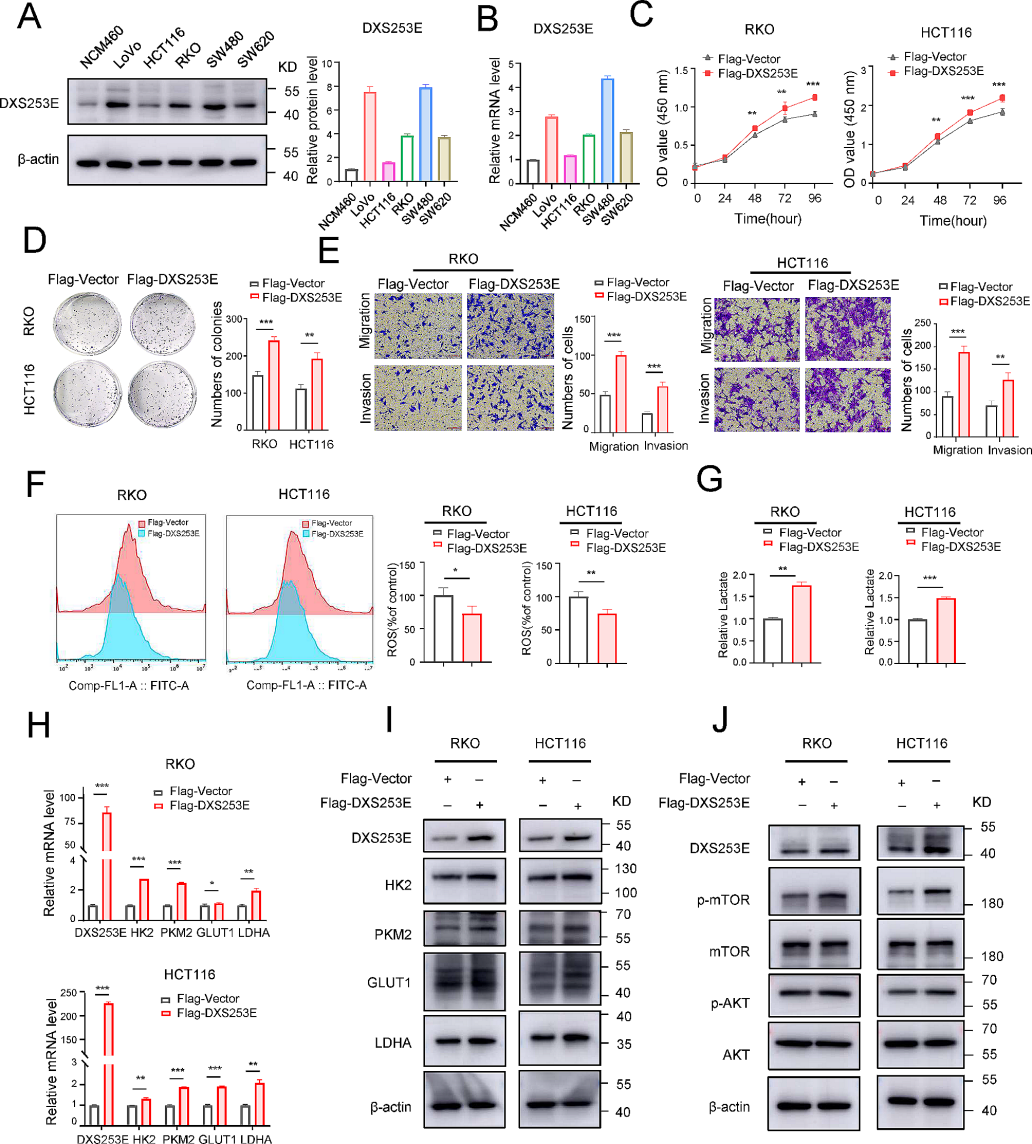
DXS253E facilitates CRC cell malignant phenotype and aerobic glycolysis via the AKT/mTOR pathway. **A** Western blot assay of DXS253E protein expression levels in NCM460, LoVo, HCT116, RKO, SW480, and SW620 cell lines. **B** qRT-PCR assay of DXS253E mRNA expression levels in normal epithelial colon cell line NCM460 and CRC cell lines. **C** DXS253E overexpression accelerates the proliferation of RKO and HCT116 cells. **D** High levels of DXS253E expression enhance colony formation in CRC cells. **E** Overexpression of DXS253E enhances the malignant progression of RKO and HCT116 cells. Bar graphs show the number of migrated or invaded cells. **F** High levels of DXS253E decrease the generation of reactive oxygen species (ROS). **G** DXS253E overexpression elevates lactate production. **H, I** DXS253E regulates the level of glycolytic genes in CRC cells, including HK2, PKM2, GLUT1, and LDHA with qRT-PCR and western blot. **J** DXS253E overexpression in CRC cells mediates the activation of the AKT/mTOR pathway. *P < 0.05, **P < 0.01, ***P < 0.001

Because DXS253E expression was elevated in the tissue of CRC patient and was linked to the poor prognosis, we explored the biological role of DXS253E in CRC. First, we examined DXS253E expression levels in different cell lines. Our western blot analyses of a normal human colon mucosal epithelial cell line (NCM460) and CRC cell lines (LoVo, RKO, HCT116, SW480, and SW620) suggested that DXS253E is highly expressed in CRC cell lines (Fig. 7A). Subsequently, the mRNA levels encoding DXS253E were obtained through qRT-PCR (Fig. 7B). We found that high DXS253E mRNA and protein levels were both positively associated with NCM460, RKO, HCT116, SW480, and SW620 cells, but not with LoVo cells. The results of a CCK-8 assay and plate cloning assay showed that DXS253E overexpression markedly enhances the proliferative ability of CRC cells in RKO and HCT116 compared with the control group (Fig. 7C, D). In addition, we used a Transwell assay to assess migration and invasion ability in RKO and HCT116. These results suggested that the migration and invasion ability of CRC cells was significantly increased when DXS253E was overexpressed (Fig. 7E). Collectively, these findings show that DXS253E can significantly enhance the malignant progression of CRC cells.

Our GSEA findings suggested that DXS253E could regulate the processes of oxidative stress in CRC. To determine whether DXS253E does regulate oxidative stress processes in CRC cells, we detected intracellular reactive oxygen species (ROS) levels using dichlorodihydrofluorescein diacetate probes. Flow cytometry results from this experiment suggested that overexpression of DXS253E decreases ROS production in CRC cells (Fig. 7F). Moreover, due to associations between the processes of aerobic glycolysis and oxidative stress, lactate production assays were performed to determine the role of DXS253E in aerobic glycolysis. Our results indicated that DXS253E overexpression significantly increases lactate production in CRC cells (Fig. 7G). Furthermore, qRT-PCR and western blot procedures were performed to assess the expression of glycolytic genes. These results revealed that the overexpression of DXS253E remarkably increases the expression of several glycolytic genes, in particular HK2, PKM2, GLUT1, and LDHA, at both the mRNA and protein level in CRC cells (Fig. 7H, I).

Increasing evidence has demonstrated that aerobic glycolysis in tumor cells is associated with the AKT/mTOR pathway [38]. Therefore, we conducted western blot analysis to evaluate the role of DXS253E in the AKT/mTOR pathway. Our results showed that DXS253E overexpression promoted the phosphorylation of AKT and mTOR in RKO and HCT116 cells (Fig. 7J). These findings raise the possibility that DXS253E increases aerobic glycolysis through the activation of the AKT/mTOR pathway in CRC cells.

## Discussion

CRC is a heterogeneous malignant tumor that exhibits a complex microenvironment [39]. This complicates efforts to understand how different genes and cell types promote disease progression. In this study, based primarily on TCGA and the GEO database, we identified that high DXS253E levels predict poor prognosis in patients with CRC. Bioinformatics analyses indicated that DXS253E is associated with genetic change and immune infiltration. Functionally, DXS253E enhances malignant phenotypes and aerobic glycolysis in CRC cells through the AKT/mTOR pathway, according to our research. Previous studies have demonstrated the clinical and prognostic significance of DXS253E in various tumors. In liver cancer, high DXS253E protein levels were observed to be significantly associated with immune cell infiltration and poor prognosis [22]. In terms of LGG, higher DXS253E expression levels contribute to programmed cell death and immune infiltration, resulting in poor prognosis of the patients with LGG [40]. Consistently, Ma et al. also found that the upregulation of DXS253E is associated with poor OS in LGG and glioblastoma [41]. Moreover, they suggested that SLC10A3 is a possible therapeutic target for LGG [41]. Furthermore, Wang et al.’s study that analyzed public databases indicates that a high DXS253E expression predicts poor prognosis in CRC patients, and they propose that it could be used as an immunotherapy target in CRC given its significant influence on the immune microenvironment [23]. Similarly, we found that CRC patients with higher DXS253E tumor expression exhibit worse outcomes in respect to both OS and DSS, suggesting that DXS253E can serve as a predictor of CRC prognosis. Therefore, DXS253E may be considered a cancer-promoting gene and could be used as a novel prognostic biomarker as well as a potential therapeutic target for CRC.

Previous studies have implicated that somatic mutations and high methylation levels within a gene are correlated with the regulation of gene expression [42, 43]. Local DNA hypermethylation analyses have potential as a diagnostic and outcome prediction tool for some cancers [44, 45]. Thus, we analyzed genetic and epigenetic alterations of the DXS253E gene in CRC. Our exploratory findings demonstrated that DXS253E genetic alterations, including mutation and amplification, can be observed in a variety of cancers. Furthermore, significant differences existed in the DXS253E gene DNA methylation levels between tumor tissues and normal tissues. Although the role of gene body methylation and the methylation of CpG sites in both introns and exons remains less characterized, we identified six CpG sites in the DXS253E gene that were correlated with the prognosis of CRC patients. Additionally, due to the reversibility of DNA methylation, hypermethylated genes could prove to be targets for the treatment of cancers.

We also investigated the DEGs between DXS253E low- and DXS253E high-expression groups in CRC to elucidate the potential biological functions and regulatory pathways of DXS253E. A previous study using GO/KEGG enrichment analysis demonstrated that DXS253E-correlated genes were related to substance transport in LGG [41]. However, we identified that DXS253E affected the transcriptome and oxidative phosphorylation metabolic pathway in CRC by conducting enrichment analyses of the GO and KEGG databases and GSEA. The difference in enrichment analyses between LGG and CRC may be due to the heterogeneity of the tumors.

Furthermore, we explored genes encoding DXS253E-related proteins and co-expressed genes in CRC tissues to identify DXS253E molecular mechanisms contributing to CRC prognosis. Consequently, we defined 53 overlapping genes between TCGA and LinkedOmics as CRC co-expressed hub genes and DEGs. Our enrichment pathway analysis showed that these hub genes were involved in vitamin metabolic processes and response to salt. Overall, these data raise the possibility that DXS253E may participate in cellular metabolism and intracellular transport and may facilitate the proliferation and metastasis of CRC by adjusting these biological processes.

Cancer progression is related to the TME, which is composed of tumor cells, mesenchymal cells, the extracellular matrix, immune cells. The infiltration of immune cells into the TME influences tumor prognosis [46]. Tian et al. confirmed significant associations between DXS253E expression and CD4^+^Tconv and tumor-infiltrating CD20^+^B cells via bioinformatics analysis and multispectral imaging techniques in HCC [22]. Similarly, a LGG study showed that DXS253E expression was associated with macrophage, CD4^+^Tconv cell, and B cell levels [40]. We consistently found that DXS253E levels were associated with the level of multiple immune-related genes and immune cell infiltration in CRC. DXS253E was significantly connected with the infiltration of NK, NK CD56bright, eosinophil, Tcm, T helper, and Th2 cells, any of which could be used as a clinical outcome indicator in CRC patients. DXS253E has dramatic effect on the partial infiltrations of immune cells in CRC tissues.

We also explored the impact of DXS253E on cell phenotype and mechanism, and identified that DXS253E overexpression can remarkably enhance the proliferation, migration, and invasion capacity of CRC cells. Our results indicate that the specific influence of DXS253E on the malignant proliferation of tumor cells may occur to some degree through effects on aerobic glycolysis via the AKT/mTOR pathway.

Although our current study provides new understanding about the relations between DXS253E expression and prognostic value in CRC patients, some limitations need further consideration. Primarily, this study used open-source datasets, which can lead to selection bias. Other independent large-scale CRC cohorts with detailed clinicopathologic characteristics will be required to further validate our findings and the application of DXS253E as a biomarker in CRC should be evaluated. Additionally, although DXS253E may offer an alternative opportunity to delay the progression of CRC, further investigation of its functions in vitro and in vivo is necessary to identify the molecular mechanism underlying DXS253E regulation of the tumor microenvironment, as well as the potential of AKT/mTOR-targeted treatment for CRC patients with high DXS253E expression.

## Conclusions

This study demonstrates that high levels of DXS253E are an independent unfavorable prognostic indicator in CRC and are notably correlated with aggressive clinical characteristics. High DXS253E levels facilitate malignant biological behavior and aerobic glycolysis in CRC cells though AKT/mTOR signaling. Our study identifies DXS253E as a potentially cancer-promoting gene and suggests its potential application as a therapeutic target for regulating the AKT/mTOR pathway in CRC.

### Electronic supplementary material

Below is the link to the electronic supplementary material.

**Supplementary Material 1: Raw data**: The whole uncropped images of the original western blots

**Supplementary Material 2: Table S1**: Clinical information for 8 CRC samples

**Supplementary Material 3: Table S2**: Relevant primer sequences

**Supplementary Material 4: Table S3**: The relationship between DXS253E expression and clinical characteristics in CRC patients with TCGA cohort

**Supplementary Material 5: Table S4**: Volcano plot for DEGs between low DXS253E and high DXS253E groups

**Supplementary Material 6: Table S5**: GO enrichment analysis of the DXS253E-associated DEGs

**Supplementary Material 7: Table S6**: KEGG enrichment analysis of the DXS253E-associated DEGs

**Supplementary Material 8: Table S7**: The DXS253E-related DEGs for GSEA analysis

**Supplementary Material 9: Table S8**: Co‐expressed genes correlated with DXS253E expression by LinkedOmics

**Supplementary Material 10: Table S9**: Overlapping genes between DEGs and co-expressed genes of DXS253E in CRC

**Supplementary Material 11: Table S10**: Enrichment analysis of overlapping genes analyzed by Metascape

## Data Availability

The raw data used to support the findings of this study are available upon reasonable request from the corresponding author. All the code has been uploaded to GitHub (https://github.com/Sulab0).

## Abbreviations

CRC: Colorectal Cancer
TCGA: The Cancer Genome Atlas
GEO: Gene Expression Omnibus
HCC: Hepatocellular Carcinoma
LGG: Low-Grade Glioma
DEGs: Differentially Expressed Gene Analysis
GSEA: Gene Function Enrichment Analysis
ROS: Reactive Oxygen Species
GO: Gene Ontology
KEGG: Kyoto Encyclopedia of Genes and Genomes
BRCA: Breast Invasive Carcinoma
CHOL: Cholangiocarcinoma
COAD: Colon Adenocarcinoma
READ: Rectum Adenocarcinoma
OS: Overall Survival
DSS: Disease-Specific Survival
TME: Tumor Microenvironment
