# Supplementary figures and images for: Integrated profiling identifies DXS253E as a potential prognostic marker in colorectal cancer
Source: Cancer Cell Int. 2024 Jun 18;24:213. doi: 10.1186/s12935-024-03403-4 (PMC11186088; doi:10.1186/s12935-024-03403-4)

A

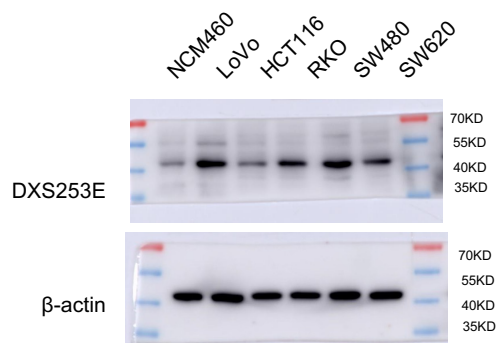

I

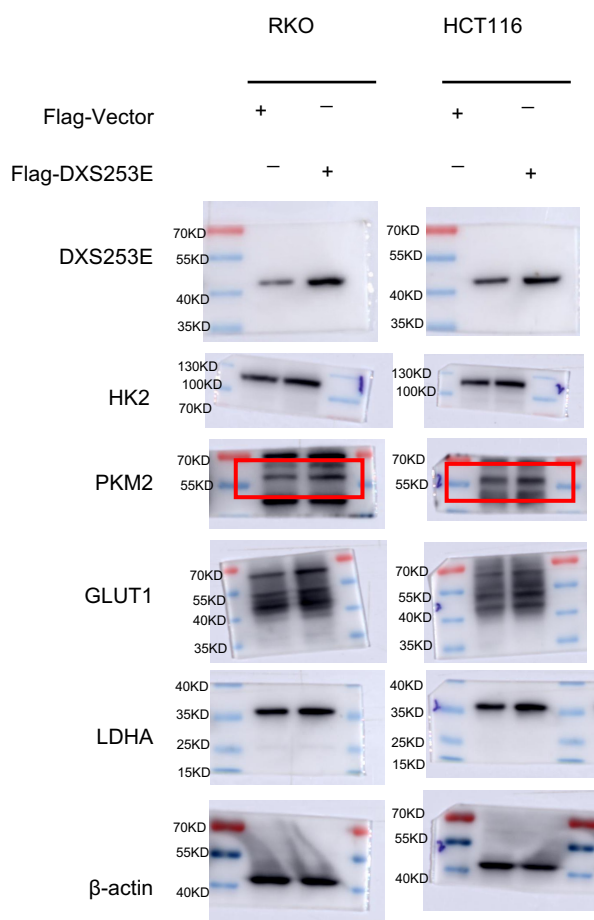

J

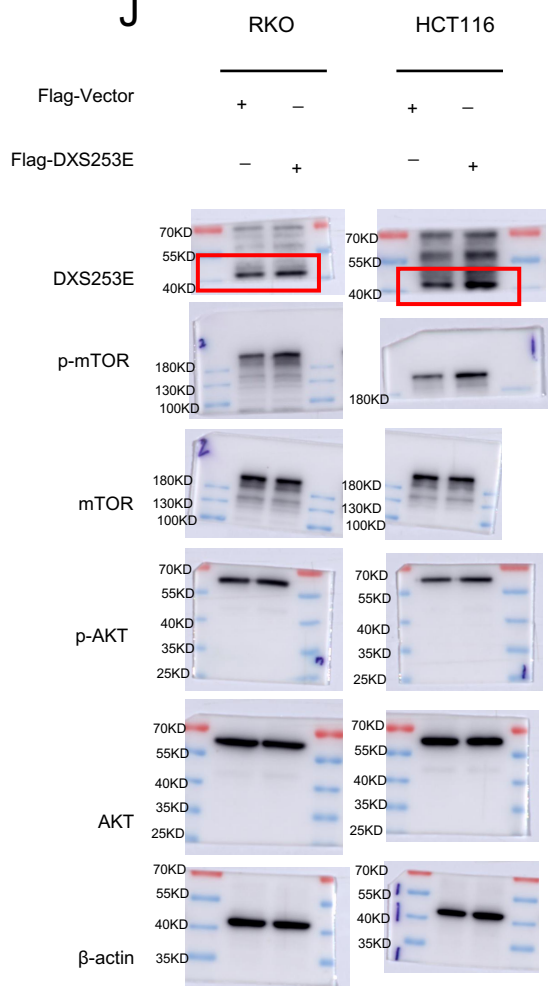

Supplement: Supplementary file 1 — Supplementary Material 1: Raw data: The whole uncropped images of the original western blots [file 12935_2024_3403_MOESM1_ESM.pdf]
