# Supplementary material for: Integrated profiling identifies DXS253E as a potential prognostic marker in colorectal cancer
Source: Cancer Cell Int. 2024 Jun 18;24:213. doi: 10.1186/s12935-024-03403-4 (PMC11186088; doi:10.1186/s12935-024-03403-4)
Supplement: Supplementary file 2 — Supplementary Material 2: Table S1: Clinical information for 8 CRC samples [file 12935_2024_3403_MOESM2_ESM.docx]

**Table S1. Clinical information for 8 CRC samples**

| Sample ID | Gender | Age | Tumor location | T stage | N stage | M stage | Pathologic stage | Differentiation grade | Preoperative treatment |
| --- | --- | --- | --- | --- | --- | --- | --- | --- | --- |
| 1 | M | 73 | Hepatic Flexure | T4a | N0 | M0 | IIA | Low | NO |
| 2 | M | 51 | Hepatic Flexure | T4a | N1 | M1a | IVA | Middle | NO |
| 3 | F | 33 | Transverse Colon | T4a | N0 | M0 | IIA | Middle | NO |
| 4 | M | 77 | Transverse Colon | T4a | N1 | M0 | IIIB | Middle | NO |
| 5 | M | 54 | Hepatic Flexure | T4b | N0 | M0 | IIB | Low | NO |
| 6 | F | 52 | Sigmoid Colon | T4a | N0 | M0 | IIA | Middle | NO |
| 7 | M | 55 | Sigmoid Colon | T2 | N0 | M0 | I | Middle | NO |
| 8 | M | 81 | Hepatic Flexure | T4b | N0 | M0 | IIB | Middle | NO |
