# Supplementary material for: Integrated profiling identifies DXS253E as a potential prognostic marker in colorectal cancer
Source: Cancer Cell Int. 2024 Jun 18;24:213. doi: 10.1186/s12935-024-03403-4 (PMC11186088; doi:10.1186/s12935-024-03403-4)
Supplement: Supplementary file 3 — Supplementary Material 3: Table S2: Relevant primer sequences [file 12935_2024_3403_MOESM3_ESM.docx]

**Table S2. Relevant primer sequences.**

| Gene | Primer sequences | |
| --- | --- | --- |
| DXS253E | Forward  Reverse | 5′- CAACAAGTGTTCGTTTGGGTG -3′  5′- CCATGAGGAAAGCGTACAAGG -3′ |
| GAPDH | Forward  Reverse | 5′- GGACTCATGACCACAGTCCATG -3′  5′- CAGGGATGATGTTCTGGAGAGC -3′ |
| HK2 | Forward  Reverse | 5′- GAGCCACCACTCACCCTACT - 3′  5′- CCAGGCATTCGGCAATGTG -3′ |
| PKM2 | Forward  Reverse | 5′- ATGTCGAAGCCCCATAGTGAA -3′  5′- TGGGTGGTGAATCAATGTCCA -3′ |
| GLUT1 | Forward  Reverse | 5′- GGCCAAGAGTGTGCTAAAGAA -3′  5′- ACAGCGTTGATGCCAGACAG -3′ |
| LDHA | Forward  Reverse | 5′- ATGGCAACTCTAAAGGATCAGC -3′  5′- CCAACCCCAACAACTGTAATCT -3′ |
