# Supplementary material for: Integrated profiling identifies DXS253E as a potential prognostic marker in colorectal cancer
Source: Cancer Cell Int. 2024 Jun 18;24:213. doi: 10.1186/s12935-024-03403-4 (PMC11186088; doi:10.1186/s12935-024-03403-4)
Supplement: Supplementary file 4 — Supplementary Material 4: Table S3: The relationship between DXS253E expression and clinical characteristics in CRC patients with TCGA cohort [file 12935_2024_3403_MOESM4_ESM.docx]

**Table S3. The relationship between DXS253E expression and clinical characteristics in CRC patients with TCGA cohort.**

| Variables | Levels | Low expression of DXS253E | High expression of DXS253E | *P*-value |
| --- | --- | --- | --- | --- |
| n |  | 322 | 322 |  |
| Gender, n (%) | Female | 154 (23.9%) | 147 (22.8%) | 0.636 |
|  | Male | 168 (26.1%) | 175 (27.2%) |  |
| Age, n (%) | ≤65 | 130 (20.2%) | 146 (22.7%) | 0.232 |
|  | >65 | 192 (29.8%) | 176 (27.3%) |  |
| T stage, n (%) | T1 | 11 (1.7%) | 9 (1.4%) | 0.941 |
|  | T2 | 56 (8.7%) | 55 (8.6%) |  |
|  | T3 | 214 (33.4%) | 222 (34.6%) |  |
|  | T4 | 38 (5.9%) | 36 (5.6%) |  |
| N stage, n (%) | N0 | 204 (31.9%) | 164 (25.6%) | **0.003** |
|  | N1 | 63 (9.8%) | 90 (14.1%) |  |
|  | N2 | 51 (8%) | 68 (10.6%) |  |
| M stage, n (%) | M0 | 247 (43.8%) | 228 (40.4%) | **0.002** |
|  | M1 | 30 (5.3%) | 59 (10.5%) |  |
| Pathologic stage, n (%) | Stage I | 58 (9.3%) | 53 (8.5%) | **0.001** |
|  | Stage II | 138 (22.2%) | 100 (16.1%) |  |
|  | Stage III | 83 (13.3%) | 101 (16.2%) |  |
|  | Stage IV | 32 (5.1%) | 58 (9.3%) |  |
| Residual tumor, n (%) | R0 | 235 (46.1%) | 233 (45.7%) | 0.086 |
|  | R1 | 1 (0.2%) | 5 (1%) |  |
|  | R2 | 13 (2.5%) | 23 (4.5%) |  |
| Perineural invasion, n (%) | No | 99 (42.1%) | 76 (32.3%) | **0.039** |
|  | Yes | 24 (10.2%) | 36 (15.3%) |  |
| Lymphatic invasion, n (%) | No | 198 (34%) | 152 (26.1%) | **< 0.001** |
|  | Yes | 91 (15.6%) | 141 (24.2%) |  |
| Neoplasm type, n (%) | Colon adenocarcinoma | 251 (39%) | 227 (35.2%) | **0.038** |
|  | Rectum adenocarcinoma | 71 (11%) | 95 (14.8%) |  |
| OS event, n (%) | Alive | 271 (42.1%) | 244 (37.9%) | **0.010** |
|  | Dead | 51 (7.9%) | 78 (12.1%) |  |
| DSS event, n (%) | Alive | 281 (45.2%) | 263 (42.3%) | **0.040** |
|  | Dead | 30 (4.8%) | 48 (7.7%) |  |

*P*-values in bold were statistically significant.
