# Supplementary material for: Integrated profiling identifies DXS253E as a potential prognostic marker in colorectal cancer
Source: Cancer Cell Int. 2024 Jun 18;24:213. doi: 10.1186/s12935-024-03403-4 (PMC11186088; doi:10.1186/s12935-024-03403-4)
Supplement: Supplementary file 6 — Supplementary Material 6: Table S5: GO enrichment analysis of the DXS253E-associated DEGs [file 12935_2024_3403_MOESM6_ESM.docx]

**Table S5. GO enrichment analysis of the DXS253E-associated DEGs.**

| Ontology | ID | Description | GeneRatio | BgRatio | pvalue | p.adjust | qvalue |
| --- | --- | --- | --- | --- | --- | --- | --- |
| BP | GO:0006334 | nucleosome assembly | 14/186 | 145/18670 | 2.03e-10 | 2.17e-07 | 2.13e-07 |
| BP | GO:0000365 | mRNA trans splicing, via spliceosome | 6/186 | 12/18670 | 7.93e-10 | 2.17e-07 | 2.13e-07 |
| BP | GO:0000353 | formation of quadruple SL/U4/U5/U6 snRNP | 6/186 | 12/18670 | 7.93e-10 | 2.17e-07 | 2.13e-07 |
| CC | GO:0015030 | Cajal body | 14/194 | 77/19717 | 2.43e-14 | 2.98e-12 | 2.63e-12 |
| CC | GO:0000786 | nucleosome | 15/194 | 107/19717 | 1.55e-13 | 9.55e-12 | 8.42e-12 |
| CC | GO:0044815 | DNA packaging complex | 15/194 | 115/19717 | 4.61e-13 | 1.89e-11 | 1.67e-11 |
| MF | GO:0033038 | bitter taste receptor activity | 8/89 | 23/17697 | 1.37e-13 | 2.17e-11 | 1.97e-11 |
| MF | GO:0008527 | taste receptor activity | 8/89 | 29/17697 | 1.17e-12 | 9.29e-11 | 8.42e-11 |
