# Supplementary material for: Integrated profiling identifies DXS253E as a potential prognostic marker in colorectal cancer
Source: Cancer Cell Int. 2024 Jun 18;24:213. doi: 10.1186/s12935-024-03403-4 (PMC11186088; doi:10.1186/s12935-024-03403-4)
Supplement: Supplementary file 7 — Supplementary Material 7: Table S6: KEGG enrichment analysis of the DXS253E-associated DEGs [file 12935_2024_3403_MOESM7_ESM.docx]

**Table S6. KEGG enrichment analysis of the SLC10A3-associated DEGs.**

| Ontology | ID | Description | GeneRatio | BgRatio | pvalue | p.adjust | qvalue |
| --- | --- | --- | --- | --- | --- | --- | --- |
| KEGG | hsa05322 | Systemic lupus erythematosus | 12/50 | 136/8076 | 2.24e-11 | 1.23e-09 | 1.13e-09 |
| KEGG | hsa05034 | Alcoholism | 13/50 | 187/8076 | 6.08e-11 | 1.67e-09 | 1.54e-09 |
| KEGG | hsa04742 | Taste transduction | 8/50 | 86/8076 | 4.43e-08 | 8.13e-07 | 7.47e-07 |
| KEGG | hsa05203 | Viral carcinogenesis | 8/50 | 204/8076 | 3.12e-05 | 4.29e-04 | 3.94e-04 |
| KEGG | hsa03040 | Spliceosome | 5/50 | 151/8076 | 0.002 | 0.025 | 0.023 |
| KEGG | hsa03013 | RNA transport | 5/50 | 186/8076 | 0.006 | 0.052 | 0.047 |
